# Psychiatric Boarding Patterns Among Publicly Insured Youths Evaluated by Mobile Crisis Teams Before and During the COVID-19 Pandemic

**DOI:** 10.1001/jamanetworkopen.2023.21798

**Published:** 2023-07-06

**Authors:** Carolina-Nicole Herrera, Rachel Oblath, Alison Duncan

**Affiliations:** 1 Department of Health, Law, Policy, and Management, Boston University School of Public Health, Boston, Massachusetts; 2 Department of Psychiatry, Boston Medical Center, Boston, Massachusetts; 3 Boston Emergency Services Team Partnership for Behavioral Health, Racial, and Social Justice, Boston Medical Center, Boston, Massachusetts; 4 Boston University Chobanian and Avedisian School of Medicine, Boston, Massachusetts

## Abstract

**Importance:**

Psychiatric boarding occurs when patients needing intensive psychiatric services who are already under clinical supervision experience delays in their admission to psychiatric facilities. Initial reports have suggested that the US had a psychiatric boarding crisis during the COVID-19 pandemic; however, little is known about the consequences of this crisis for publicly insured youths.

**Objective:**

To estimate pandemic-associated changes in psychiatric boarding rates and discharge modalities for people aged 4 to 20 years who accessed psychiatric emergency services (PES) through a mobile crisis team (MCT) evaluation and were covered by Medicaid or health safety net programs.

**Design, Setting, and Participants:**

This retrospective cross-sectional study used data from the MCT encounters of a multichannel PES program in Massachusetts. A total of 7625 MCT-initiated PES encounters with publicly insured youths who lived in Massachusetts between January 1, 2018, and August 31, 2021, were assessed.

**Main Outcomes and Measures:**

Encounter-level outcomes (psychiatric boarding status, repeat visits, and discharge disposition) during a prepandemic period (January 1, 2018, to March 9, 2020) were compared with outcomes during a pandemic period (March 10, 2020, to August 31, 2021). Descriptive statistics and multivariate regression analysis were used.

**Results:**

Among 7625 MCT-initiated PES encounters, the mean (SD) age of publicly insured youths was 13.6 (3.7) years; most youths identified as male (3656 [47.9%]), were of Black race (2725 [35.7%]) or Hispanic ethnicity (2708 [35.5%]), and spoke English (6941 [91.0%]). During the pandemic period, the mean monthly boarding encounter rate was 25.3 percentage points higher than the prepandemic period. After adjustment for covariates, the odds of an encounter resulting in boarding doubled during the pandemic (adjusted odds ratio [AOR], 2.03; 95% CI, 1.82-2.26; *P* < .001), and boarding youths were 64% less likely to be discharged to inpatient psychiatric care (AOR, 0.36; 95% CI, 0.31-0.43; *P* < .001). Publicly insured youths who boarded during the pandemic had significantly higher rates of 30-day readmissions (incidence rate ratio, 2.17; 95% CI, 1.88-2.50; *P* < .001). Boarding encounters during the pandemic were significantly less likely to end in discharge to inpatient psychiatric units (AOR, 0.36; 95% CI, 0.31-0.43; *P* < .001) or community-based acute treatment facilities (AOR, 0.70; 95% CI, 0.55-0.90; *P* = .005).

**Conclusions and Relevance:**

In this cross-sectional study, publicly insured youths were more likely to experience psychiatric boarding during the COVID-19 pandemic and, if boarding, were less likely to transfer to a 24-hour level of care. These findings suggest that psychiatric service programs for youths were not prepared to support the levels of acuity and demand that emerged from the pandemic.

## Key Points

**Question:**

Was the COVID-19 pandemic associated with changes in psychiatric boarding among publicly insured youths who were initially evaluated by a mobile crisis team?

**Findings:**

In this cross-sectional study of 7625 psychiatric emergency services encounters, publicly insured youths evaluated by mobile crisis teams were 2 times more likely to experience psychiatric boarding, and boarding youths were 64% less likely to be discharged to inpatient psychiatric care during the pandemic.

**Meaning:**

These findings suggest that psychiatric service programs were not prepared to support the levels of acuity and demand from youths that emerged during the pandemic.

## Teaser

This cross-sectional study assesses COVID-19 pandemic–associated changes in psychiatric boarding rates and discharge modalities among publicly insured youths who accessed psychiatric emergency services through a mobile crisis team evaluation.

## Introduction

Psychiatric boarding occurs when patients who accessed psychiatric emergency services (PES) require treatment in an inpatient psychiatric unit (IPU) or community-based acute treatment (CBAT) facility but experience administrative delays in their transfer and remain under clinical supervision in suboptimal care.^1^ An ongoing goal in PES has been to reduce psychiatric boarding (hereafter, boarding).^2,3,4^

**Figure 1.**
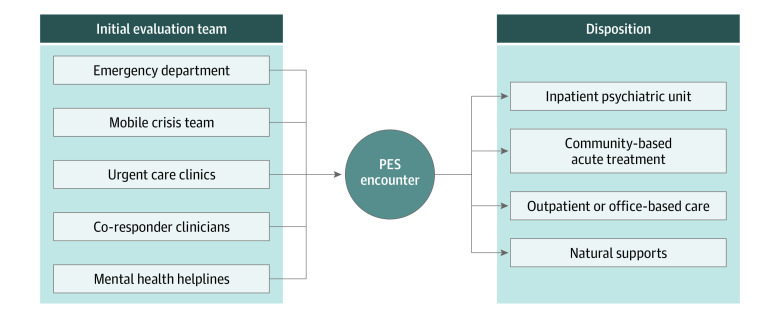
Pathways Into and Out of Psychiatric Emergency Service (PES)

Psychiatric emergency services seek to stabilize people experiencing acute psychiatric crises and connect them with other care settings in which they can receive appropriate and timely mental health treatment (Figure 1).^5^ Stabilization procedures include a psychiatric evaluation, provision of a safe environment, 24-hour supervision, and medication management.^6^ After stabilization, patients may be discharged without referral (ie, discharged to natural supports [NS], defined as individuals who provide unpaid assistance with care needs) or to outpatient care. Patients at higher risk may be transferred to unlocked voluntary 24-hour CBAT facilities. Patients with the most severe symptoms, including psychosis, homicidal ideation, and suicidality, may be transferred to locked IPUs.^7,8,9^

Typically, boarding occurs when the volume of patients who need PES treatment in an IPU or CBAT facility is greater than the number of available beds. Boarding is not a therapeutic intervention and has been associated with lower quality of care and higher health care costs.^10,11,12,13^ Patients who board for prolonged periods may experience worsening of symptoms. Underinvestment, increasing patient loads, and staffing difficulties contributed to a national shortage in psychiatric beds before the onset of the pandemic.^14,15,16^ Findings of a previous study^17^ of boarding suggest that rates have been increasing since 2001, particularly among publicly insured youths.

Initial reports suggested the boarding crisis worsened during the pandemic.^18,19,20^ Multiple studies reported declines in medical emergency service use in 2020.^21,22^ Growing evidence suggests that after the initial lockdowns, PES units experienced rapid returns to prepandemic encounter rates and a surge in psychiatric emergency department (ED) visits among adolescents at risk of suicide.^22,23,24^ One study^25^ hypothesized that IPU capacity would be substantially altered by COVID-19–related restrictions, such as the separation of patients by COVID-19 status and reductions in group spaces, which would reduce the number of available psychiatric beds. A study of children who presented to community hospital ED settings for mental health care found children’s time in the ED lengthened during the COVID-19 pandemic.^26^

Publicly insured youths with psychiatric illnesses are among the most vulnerable populations in the US; however, few studies have examined to what extent the pandemic has been associated with the use of PES among publicly insured youths. By the end of the pandemic, more than 50% of children in the US were covered by public insurance.^27,28^ Moreover, Medicaid is an important source of insurance for children and young adults with behavioral health conditions.^29^ National estimates of PES rates during the COVID-19 pandemic comingled the publicly and privately insured children’s populations despite research among adults suggesting that people with Medicaid coverage generally have higher rates of PES use than people with private insurance.^18,30^

Another limitation of pandemic research on PES has been its focus on ED settings. Efforts to reduce overcrowding in EDs while expanding access to PES have led to innovations in the delivery of 24-hour acute mental health care. People access PES through multiple channels, including EDs, urgent care clinics, and mobile crisis teams (MCTs) (Figure 1). Psychiatric urgent care clinics, MCTs, and police co-responder programs are a growing source of PES.^31,32^ Psychiatric MCTs have existed since the early 1990s but were only recognized as best practice for psychiatric systems in the 2000s.^33,34,35,36,37^ These teams deliver easily accessible targeted psychiatric triage services by responding to emergencies in the community at the requests of families, schools, and police departments. Similar to patients evaluated in the ED, those evaluated by MCTs may require admission to 24-hour levels of care and, depending on the availability of beds, may spend hours to days boarding. To our knowledge, previous research has not examined boarding among publicly insured youths evaluated by MCTs. As MCT programs expand nationwide, it is important to understand to what extent populations evaluated by MCTs are likely to require intensive treatment and how existing systems incorporate MCTs into PES workflow.

In this study, we sought to characterize encounter-level boarding of publicly insured youths who were evaluated by MCTs between 2018 and 2021. We aimed to estimate the extent to which the pandemic was associated with changes in boarding rates, repeat visits, and discharge modalities among publicly insured youths. We hypothesized that (1) the pandemic would be associated with boarding rates, repeat visits, and discharge modalities for publicly insured youths evaluated by MCTs who boarded and (2) the pandemic would be associated with increases in boarding length of stay (LOS) for publicly insured youths evaluated by MCTs.

## Methods

We retrospectively examined data from 7625 encounters with publicly insured patients aged 4 to 20 years evaluated by MCTs at an urban PES program between January 1, 2018, and August 31, 2021. The MCTs were part of the Boston Emergency Services Team (BEST), an urban PES program that serves 5 cities in Massachusetts: Boston, Cambridge, New Bedford, Revere, and Somerville. All study procedures were approved by the Boston Medical Center Institutional Review Board. A waiver of informed consent was granted because the data in this study were obtained from existing medical records. This study followed the Strengthening the Reporting of Observational Studies in Epidemiology (STROBE) reporting guideline for cross-sectional studies.^38^

### Setting

Data for this study were obtained from the BEST electronic health record (EHR),^31^ which includes patient demographic characteristics, encounter characteristics, disposition, and psychiatric disorder information. The BEST MCTs provide services in community locations, including schools, homes, and public places. These MCTs deliver office-based care through 3 PES urgent care centers and provide evaluations for patients in nonaffiliated EDs.^31^

During the COVID-19 pandemic, BEST MCTs continued to provide around-the-clock care and did not turn any patients away due to COVID-19 infection. The BEST MCTs offered telehealth evaluations with patients who had symptoms of COVID-19 or who did not want an in-person evaluation. Of 13 795 BEST encounters with publicly insured youths, 10 560 (75.6%) began with an MCT evaluation.

### Data

From the BEST EHR, we identified all encounters with publicly insured people aged 4 to 20 years in which the initial evaluation was performed by an MCT. Any encounters occurring within 6 hours of a previous encounter were combined with that previous encounter. Patients were considered to be psychiatrically boarding if they required 24-hour psychiatric care and an IPU or CBAT bed was unavailable within 6 hours of the disposition decision.

Among 10 560 MCT-initiated encounters, we excluded 2935 potentially eligible encounters due to missing demographic data and boarding status. The final analytic sample comprised 7625 MCT-initiated encounters. Sensitivity tests suggested the proportion of encounters that involved boarding did not differ between encounters with and without complete demographic data (eTable 1 in Supplement 1). Demographic data and discharge outcomes of encounters that were missing boarding status were most similar to those of the nonboarding study sample (eTable 2 in Supplement 1). Missing demographic information is a common issue with EHR data and may result from youth and families declining to or being unable to provide information or clinicians failing to collect or record information.^39,40^ The rate of missing race and ethnicity data in our study was lower than rates of missing data from other EHR data sets used in scientific research.^40^ Missing boarding status may be a product of varying comprehensiveness in documenting clinical information by administrators and clinicians, which is also a common issue with EHR data.^41,42^

#### Variables of Interest

The COVID-19 pandemic period was the treatment exposure and defined as March 10, 2020, through August 31, 2021. Encounter-level outcomes during this period were compared with those during the prepandemic period of January 1, 2018, to March 9, 2020. Outcomes of interest were boarding status, repeat visits, and discharge disposition. Boarding status was a binary outcome associated with a specific encounter. A visit was considered a repeat if the subsequent evaluation was performed by any BEST clinician within 30 days of the previous visit. Discharge disposition had 4 outcomes: IPU, CBAT facility, outpatient care, or NS. Covariates included demographic, encounter, and diagnostic data. Demographic data included age, gender identity, race and ethnicity (Asian/Pacific Islander, Black, Hispanic, White, and other [which included those who selected “other” or indicated American Indian/Alaska Native]), and zip code of residence. Race and ethnicity data were relevant to the study outcome because of known racial and ethnic disparities in mental health care.^43^ Encounter data included referral source, location of encounter, and primary diagnosis. Primary psychiatric diagnoses were organized into categories using *International Classification of Diseases, Tenth Revision* codes and *Diagnostic and Statistical Manual of Mental Disorders* (Fifth Edition) criteria.^32^

A secondary outcome of interest was boarding LOS, which was estimated by counting the hours between bed search start and end times and dividing that number by 24 to estimate the number of days. A total of 3624 encounters had a boarding flag; of those, only 1752 (48.3%) had bed search time stamps. We therefore conducted boarding LOS analyses using that subset of data. Sensitivity tests indicated that encounters without bed search data were more likely to result in discharge to outpatient care and less likely to result in discharge to IPUs than encounters with bed search data (eTable 3 in Supplement 1). For this reason, caution should be used when interpreting the boarding LOS findings.

### Statistical Analysis

We were interested in how the pandemic was associated with differences in (1) encounter characteristics, (2) odds of boarding, (3) number of visits per publicly insured youth, (4) odds of disposition modality, and (5) changes to boarding LOS. Statistical differences for categorical variables were estimated using χ^2^ analysis. Mean differences for continuous and normalized variables were estimated using a 2-sided Welch *t* test. We estimated pandemic-related changes in the incidence rates of visits and repeat visits by boarding status using Poisson regression models with an exposure of days to account for differences in the length of time in the 2 periods. The likelihoods of boarding and discharge outcomes were estimated using logistic regression analysis. We used log-linear regression analysis to estimate boarding LOS for the subset of the boarding sample with available data (n = 1752). Regression covariates included patient demographic characteristics (age, gender identity, race and ethnicity, and zip code of residence) and encounter characteristics (primary diagnosis, referral source, and evaluation location). Sensitivity analyses were performed to confirm model fit. All analyses were conducted using Stata software, version 16.2 (StataCorp LLC). The threshold for statistical significance was 2-sided *P* = .01.

## Results

Among 7625 PES encounters during the prepandemic and pandemic periods, the mean (SD) age of publicly insured youths was 13.6 (3.7) years; 3656 (47.9%) identified as male, 3606 (47.3%) as female, and 363 (4.8%) as transgender or nonbinary. A total of 289 encounters (3.8%) were among Asian/Pacific Islander youths, 2725 (35.7%) among Black youths, 2708 (35.5%) among Hispanic youths, 1485 (19.5%) among White youths, and 418 (5.5%) among youths of other races and/or ethnicities. Most encounters were among English-speaking youths (6941 [91.0%]) (Table 1). Some demographic data changed during the COVID-19 pandemic. The share of encounters with transgender or nonbinary youths significantly increased during the pandemic (nonboarding encounters: odds ratio [OR], 1.69 [95% CI, 1.18-2.43; *P* < .001]; boarding encounters: OR, 1.64 [95% CI, 1.25-2.14; *P* < .001]). Compared with the prepandemic period, boarding encounters during the pandemic were less likely to have a primary diagnosis of stress or adjustment disorder (OR, 0.75; 95% CI, 0.65-0.85; *P* < .001) and more likely to have a primary diagnosis of eating disorder (OR, 3.03; 95% CI, 1.59-5.77; *P* < .001) or developmental delay (OR, 1.37; 95% CI, 1.16-1.62; *P* < .001).

**Table 1.** Encounter and Patient Demographic Characteristics, 2018-2021

Characteristic	Encounters, No. (%)				Change from prepandemic to pandemic period, OR (95% CI)^b^	
	All (N = 7625)	Comparison by boarding status^a^				
		Nonboarding (n = 4001)	Boarding (n = 3624)	*P* value^c^	Nonboarding	Boarding
Period						
Prepandemic^d^	4969 (65.2)	3010 (60.6)	1959 (39.4)	<.001	NA	NA
Pandemic^e^	2656 (34.8)	991 (37.3)	1665 (62.7)			
Age, mean (SD), y	13.6 (3.7)	13.0 (3.7)	14.3 (3.6)	<.001	NA	NA
Race and ethnicity						
Asian/Pacific Islander	289 (3.8)	135 (46.7)	154 (53.3)	<.001	1.34 (0.92-1.95)	1.06 (0.77-1.47)
Black	2725 (35.7)	1481 (54.3)	1244 (45.7)		0.97 (0.83-1.12)	0.96 (0.84-1.10)
Hispanic	2708 (35.5)	1564 (57.8)	1144 (42.2)		0.96 (0.83-1.12)	0.89 (0.78-1.03)
White	1485 (19.5)	592 (39.9)	893 (60.1)		1.03 (0.84-1.25)	1.24 (1.07-1.45)
Other^f^	418 (5.5)	229 (54.8)	189 (45.2)		1.06 (0.78-1.44)	0.82 (0.61-1.10)
Gender identity						
Male	3656 (47.9)	1885 (51.6)	1771 (48.4)	<.001	1.32 (1.14-1.52)	1.08 (0.94-1.23)
Female	3606 (47.3)	1983 (55.0)	1623 (45.0)		0.70 (0.61-0.81)	0.83 (0.72-0.94)
Transgender or nonbinary	363 (4.8)	133 (36.6)	230 (63.4)		1.69 (1.18-2.43)	1.64 (1.25-2.14)
Language						
Does not speak English	684 (9.0)	380 (55.6)	304 (44.4)	.09	1.27 (1.00-1.60)	1.19 (0.94-1.51)
Speaks English	6941 (91.0)	3621 (52.2)	3320 (47.8)		0.79 (0.62-1.00)	0.84 (0.66-1.06)
Primary mental health condition						
Stress or adjustment disorder	3595 (47.1)	2242 (62.4)	1353 (37.6)	<.001	0.91 (0.79-1.05)	0.75 (0.65-0.85)
Psychotic disorder	365 (4.8)	95 (26.0)	270 (74.0)		1.03 (0.64-1.64)	0.85 (0.66-1.09)
Personality disorder	41 (0.5)	18 (43.9)	23 (56.1)		0.18 (0.02-1.34)	0.76 (0.33-1.75)
Mood disorder	1880 (24.7)	750 (39.9)	1130 (60.1)		1.02 (0.85-1.23)	1.04 (0.90-1.20)
Anxiety disorder	306 (4.0)	191 (62.4)	115 (37.6)		0.90 (0.64-1.28)	1.39 (0.96-2.02)
Developmental delay	1326 (17.4)	677 (51.1)	649 (48.9)		1.19 (0.98-1.43)	1.37 (1.16-1.62)
Eating disorder	58 (0.8)	12 (20.7)	46 (79.3)		2.18 (0.69-6.87)	3.03 (1.59-5.77)
Other	54 (0.7)	16 (29.6)	38 (70.4)		1.38 (0.48-3.99)	1.31 (0.69-2.49)
Discharge modality						
IPU	2251 (29.5)	225 (10.0)	2026 (90.0)	<.001	0.60 (0.42-0.86)	0.41 (0.36-0.47)
CBAT facility	491 (6.4)	70 (14.3)	421 (85.7)		0.83 (0.46-1.47)	0.65 (0.52-0.80)
Outpatient care	2899 (38.0)	1981 (68.3)	918 (31.7)		1.18 (1.02-1.36)	3.78 (3.21-4.43)
NS	1984 (26.0)	1725 (86.9)	259 (13.1)		0.95 (0.82-1.09)	1.31 (1.01-1.68)

Abbreviations: CBAT, community-based acute treatment; IPU, inpatient psychiatric unit; NA, not applicable; NS, natural supports; OR, odds ratio.

^a^ Percentages were calculated across rows.

^b^ Odds ratios during the COVID-19 pandemic were assessed using simple bivariate logit regression models without covariates.

^c^ Tests of significance between boarding and nonboarding samples were performed using a Pearson χ^2^ test, with the exception of the age variable, for which analysis of variance was used.

^d^ January 1, 2018, to March 9, 2020.

^e^ March 10, 2020, to August 31, 2021.

^f^ Included those who selected “other” or indicated Americn Indian/Alaska Native.

### Boarding Status

**Figure 2.**
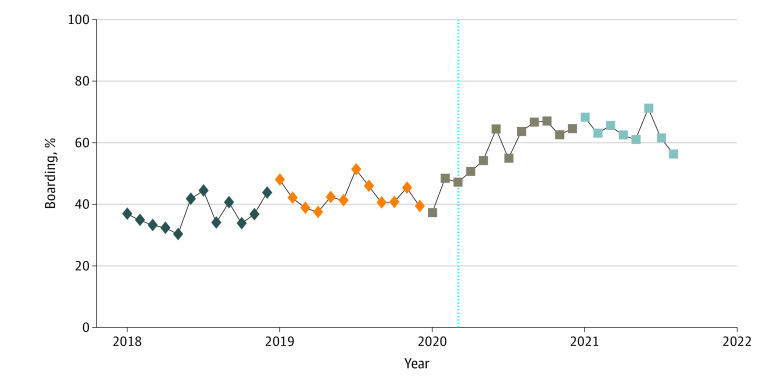
Unadjusted Monthly Psychiatric Boarding Rates for Encounters Among Publicly Insured Youths Evaluated by Mobile Crisis Teams, 2018-2021 The vertical dashed line indicates the end of the prepandemic period (January 1, 2018, to March 9, 2020) and the beginning of the pandemic period (March 10, 2020, to August 31, 2021). Each color represents a year.

During the pandemic, the mean monthly rate of boarding encounters increased by 25.3 percentage points, from 37.4% to 62.7% (Figure 2). The mean number of visits per publicly insured youths who boarded increased from 0.75 (95% CI, 0.71-0.79) in the prepandemic period to 1.28 (95% CI, 1.19-1.36) in the pandemic period (Table 2). During the pandemic, the adjusted number of encounters per publicly insured youths who boarded was approximately 1.6 times higher (incidence rate ratio, 1.56; 95% CI, 1.46-1.67; *P* < .001) and 30-day readmissions were approximately 2.2 times higher (incidence rate ratio, 2.17; 95% CI, 1.88-2.50; *P* < .001) compared with before the pandemic (Table 3). After adjustment for covariates, the odds of an encounter resulting in boarding doubled during the pandemic (adjusted OR [AOR], 2.03; 95% CI, 1.82-2.26; *P* < .001).

**Table 2.** Unadjusted Rates Among Boarding Encounters Before and During COVID-19 Pandemic

Outcome	Rate, mean (95% CI)	
	Before pandemic^a^	During pandemic^b^
Mean visits per patient evaluated by MCT, No.		
Visits	2.48 (2.38-2.57)	2.48 (2.35-2.60)
30-d Readmissions to any BEST location	0.69 (0.63-0.75)	0.95 (0.85-1.05)
Boarding in any BEST location	0.75 (0.71-0.79)	1.28 (1.19-1.36)
Discharge modality after boarding, No. (%)		
IPU	1290 (65.8)	736 (44.2)
CBAT facility	267 (13.6)	154 (9.2)
Outpatient care	278 (14.2)	640 (38.4)
NS	124 (6.3)	135 (8.1)
Mean boarding LOS, d		
IPU discharge	1.40 (1.27-1.51)	1.99 (1.73-2.25)
CBAT discharge	2.20 (1.90-2.49)	3.15 (2.32-3.98)
Outpatient discharge	3.34 (2.77-3.94)	5.31 (4.57-6.05)
NS discharge	2.48 (1.92-3.04)	2.44 (1.90-2.99)

Abbreviations: BEST, Boston Emergency Services Team; CBAT, community-based acute treatment; IPU, inpatient psychiatric unit; LOS, length of stay; MCT, mobile crisis team; NS, natural supports.

^a^ January 1, 2018, to March 9, 2020.

^b^ March 10, 2020, to August 31, 2021.

**Table 3.** Covariate-Adjusted Regression Analysis of Changes During COVID-19 Pandemic by Boarding Status

Outcome during pandemic^a^	Change from prepandemic to pandemic period
Overall boarding, AOR (95% CI)	2.03 (1.82 to 2.26)
Visits per patient, IRR (95% CI)	
Nonboarding	1.39 (1.34 to 1.44)
Boarding	1.56 (1.46 to 1.67)
30-d Readmissions to any BEST location per patient, IRR (95% CI)	
Nonboarding	1.22 (0.93 to 1.62)
Boarding	2.17 (1.88 to 2.50)
Discharge modality, AOR (95% CI)	
IPU	
Nonboarding	0.53 (0.37 to 0.76)
Boarding	0.36 (0.31 to 0.43)
CBAT facility	
Nonboarding	0.73 (0.35 to 1.53)
Boarding	0.70 (0.55 to 0.90)
Outpatient care	
Nonboarding	1.13 (0.94 to 1.36)
Boarding	3.61 (3.01 to 4.32)
NS	
Nonboarding	1.02 (0.84 to 1.23)
Boarding	1.45 (1.04 to 2.00)
Mean boarding LOS, β (95% CI)	
Overall	0.57 (0.46 to 0.69)
IPU discharge	0.59 (0.46 to 0.72)
CBAT discharge	0.28 (−0.08 to 0.63)
Outpatient discharge	0.32 (−0.02 to 0.67)
NS discharge	0.19 (−0.26 to 0.64)

Abbreviations: AOR, adjusted odds ratio; BEST, Boston Emergency Services Team; CBAT, community-based acute treatment; IPU, inpatient psychiatric unit; IRR, incidence rate ratio; LOS, length of stay; NS, natural supports.

^a^ March 10, 2020, to August 31, 2021.

### Disposition After Boarding

The share of boarding encounters ending with discharge to IPUs and CBAT facilities declined during the COVID-19 pandemic. Discharges to IPUs decreased from 65.8% of encounters in the prepandemic period to 44.2% of encounters in the pandemic period (difference, −21.6 percentage points) (Table 2). Discharges to CBAT facilities decreased from 13.6% of encounters in the prepandemic period to 9.3% of encounters in the pandemic period (difference, −4.4 percentage points). The odds of an IPU discharge decreased during the pandemic (AOR, 0.53; 95% CI, 0.37-0.76; *P* < .001) (Table 3). During the pandemic, boarding encounters were 64% less likely to discharge to IPUs (AOR, 0.36; 95% CI, 0.31-0.43; *P* < .001) and 30% less likely to discharge to CBAT facilities (AOR, 0.70; 95% CI, 0.55-0.90; *P* = .005). Boarding encounters were 261% more likely to discharge to outpatient care (AOR, 3.61; 95% CI, 3.01-4.32; *P* < .001) and 45% more likely to discharge to NS (AOR, 1.45; 95% CI, 1.04-2.00; *P* < .001).

### Changes in Boarding LOS

Boarding LOS information was only available for 1752 youths (48.3%). During the pandemic, boarding encounters were significantly longer (Table 2). After adjustments for covariates, boarding LOS increased by approximately 0.60 days for discharges to IPUs (β = 0.59 days; 95% CI, 0.46-0.72 days; *P* < .001) (Table 3). Adjusted boarding LOS did not significantly change for other discharge modalities.

## Discussion

This retrospective cross-sectional study found that publicly insured youths evaluated by MCTs were twice as likely to experience boarding and 64% less likely to experience discharge to IPUs during the COVID-19 pandemic. Publicly insured youths who boarded during the pandemic had 2.2 times the rate of 30-day readmissions than those who boarded before the pandemic. Among the small subsample of publicly insured youths for whom boarding LOS data were available, waiting times for IPUs and CBAT facilities significantly increased. These findings suggested that during the pandemic, publicly insured youths in our study were more likely to board and less likely to be discharged to the standard of care for which they were boarding.

High boarding rates suggested that acuity among publicly insured youths may have increased during the pandemic. To our knowledge, there were no changes to the criteria for PES admissions, no changes in training, and no changes in diversion practices among BEST MCTs during the pandemic. On the other hand, patients may have chosen to avoid PES due to fear of COVID-19 infection, which would have left only patients with the most severe psychiatric conditions in PES programs. While this study was not able to determine how many encounters were associated with self-harm, other studies^44,45,46^ found that the stringency of COVID-19–related lockdowns was associated with a 5-fold increase in self-harm presentations by youths. Youths were more vulnerable and more susceptible to abuse and/or neglect during the pandemic.^44,47,48^ Pandemic-related trauma is believed to have increased the likelihood of psychiatric symptoms and self-harm events among youths.^18,21,23,48^

High boarding rates coupled with lower discharge rates to IPUs and CBAT facilities suggested an insufficient number of IPU and CBAT beds were available during the pandemic. Some researchers had hypothesized that efforts to reduce COVID-19 infections would reduce available IPU and CBAT beds.^25^ National reporting on the health care workforce suggests that hospitals, IPUs, and CBAT facilities experienced a series of staffing shortages that correlated with surges in local COVID-19 infections.^49^

More publicly insured youths boarded and then discharged without intensive intervention. Longer wait times may have allowed some boarding youths to stabilize in PES programs, which could make office-based care with safety plans a better alternative. For a boarding youth to be discharged to office-based care, clinicians must decide that the youth (1) has stabilized, (2) can return to the home setting with a safety plan, and (3) no longer meets the criteria for a 24-hour level of care. The ways in which publicly insured youths who received IPU or CBAT care fared relative to those who were discharged to outpatient care or NS could not be determined from this study’s data. Further investigation of postdischarge outcomes among youths who board and discharge to outpatient care or NS is warranted.

Higher rates of 30-day readmissions among publicly insured youths who boarded suggested that some youths were unable to stabilize after discharge from PES programs. The lack of stabilization may be due to preexisting issues of mental health care access or problems accessing regular care. Some studies^26,45,50^ have hypothesized that the closure of some in-person health care services and school-based psychiatric services reduced access to regular care. A less explored but equally problematic issue was the closure of in-person partial hospitalization programs (PHPs).^51^ These programs are a form of intensive outpatient care, providing daily therapy to youths who can discharge to home on a safety plan. This approach provides a substantial level of care (3-5 hours of daily therapy) while allowing youths to continue to live at home and, in some cases, attend school. While some PHPs converted to telemedicine during the pandemic, it is unknown how accessible or effective these virtual PHPs were.^52^ It is possible that the closure of in-person PHPs may have played a role in the demand for beds by contributing to the destabilization of previously stabilized youths. Additional work on the postdischarge experiences of publicly insured youths who received PES during the pandemic is warranted to understand whether non-PES systems were sufficiently resilient during that period and what measures can be taken to reduce the risk of undertreated psychiatric illness among publicly insured youths during future public health crises.

Increases in boarding also placed additional stress on PES systems. During boarding, publicly insured youths received daily psychiatric examinations. While brief, these examinations required clinicians to speak with guardians and youths, consult with other clinicians and outpatient practitioners, and complete additional paperwork. With 62.7% of the encounters with publicly insured youths during the pandemic resulting in boarding, the PES teams were likely in a workforce crisis from June 1, 2020, through August 31, 2021. The strain from the pandemic among PES staff is beginning to be investigated,^53^ but it is reasonable to expect that future research into the consequences of the pandemic for the PES workforce is likely to find higher levels of burnout, errors, and turnover.

The pandemic required PES programs to provide more than triage, stabilization, and transfer to appropriate care. Increases in boarding among publicly insured youths created additional burdens for MCTs and broader PES systems. Increases in mental health problems among youths have been persistent even as the pandemic has subsided,^54^ suggesting that demand for PES among publicly insured youths may remain high into the near future. Without additional resources, the boarding crisis may persist. Policy makers should consider expanding community care to make the mental health system more robust. Increases in IPU and CBAT beds may help reduce boarding. Until health care systems adjust to pandemic-based increases in demand and acuity, PES programs may have difficulty connecting youths in crisis to timely and beneficial treatment.

### Limitations

This study has several limitations. The study only examined care provided by MCTs in 1 urban PES system; therefore, the findings may not be generalizable to other populations. The BEST MCT performed its work directly in the community and within EDs and remotely via telehealth, which may be a broader scope than MCTs in other areas. However, the breadth of the BEST MCT allowed this analysis to examine the overall PES experience of publicly insured youths during the pandemic. Our data were obtained from EHRs that do not include discrete indicators of homicidal ideation, suicidal behaviors, self-harm, or acute injury. The data also do not specify whether a youth boarded at home, an ED, a medical unit, or a hospital hallway. Future research is needed to understand the qualitative differences between boarding locations, particularly for publicly insured youths and persons evaluated by MCTs. Boarding LOS information was only available for 48.3% of this study’s sample and should be interpreted cautiously.

## Conclusions

This cross-sectional study found that publicly insured youths were more likely to experience psychiatric boarding during the COVID-19 pandemic, and those who were boarding were less likely to transfer to a 24-hour level of care. These findings suggest that psychiatric service programs for youths were not prepared to support the levels of acuity and demand that emerged from the pandemic.

## Notes

Supplement 1. **eTable 1.** Missing Demographic Data Compared With Study Sample **eTable 2.** Missing Boarding Status Compared With Known Boarding Status **eTable 3.** Missing Bed Search Data Compared With Known Bed Search Data

Supplement 2.
Data Sharing Statement

## References

[1] McEnany FB, Ojugbele O, Doherty JR, McLaren JL, Leyenaar JK. Pediatric mental health boarding. Pediatrics. 2020;146(4):e20201174. doi:10.1542/peds.2020-1174 32963020

[2] Parwani V, Tinloy B, Ulrich A, . Opening of psychiatric observation unit eases boarding crisis. Acad Emerg Med. 2018;25(4):456-460. doi:10.1111/acem.13369 29266537

[3] Delayed and deteriorating: serious mental illness and psychiatric boarding in emergency departments. Treatment Advocacy Center. November 2019. Accessed January 29, 2023. https://www.treatmentadvocacycenter.org/component/content/article/220-learn-more-about/4225-delayed-and-deteriorating

[4] Bender D, Pande N, Ludwig M; The Lewin Group. A literature review: psychiatric boarding. Office of the Assistant Secretary for Planning and Evaluation, US Dept of Health and Human Services. October 28, 2008. Accessed May 15, 2022. https://aspe.hhs.gov/reports/literature-review-psychiatric-boarding-0

[5] Gutherz C, Baron S. Why patients with primary care physicians use the emergency department for non-urgent care. *Einstein Q J Biol Med*. 2001;18:171-176.

[6] Levine BH, Najara JE. Child and adolescent emergency psychiatry. In: Riba MB, Ravindranath D, Winder GS, eds. *Clinical Manual of Emergency Psychiatry*. 2nd ed. American Psychiatric Association; 2016:223-252.

[7] Boege I, Fegert JM. Debate: quantity of impatient beds and quality of child psychiatric and psychotherapeutic care provision—a German perspective. Child Adolesc Ment Health. 2021;26(2):169-170. doi:10.1111/camh.12468 33797150

[8] Stanton J. Debate: the role of inpatient units is to support community care. Child Adolesc Ment Health. 2021;26(2):184-185. doi:10.1111/camh.12462 33786964

[9] Cotgrove A, Northover G. Debate: the future of inpatient units—do we need them? Child Adolesc Ment Health. 2021;26(2):178-179. doi:10.1111/camh.12466 33829618

[10] Pearlmutter MD, Dwyer KH, Burke LG, Rathlev N, Maranda L, Volturo G. Analysis of emergency department length of stay for mental health patients at ten Massachusetts emergency departments. Ann Emerg Med. 2017;70(2):193-202. doi:10.1016/j.annemergmed.2016.10.005 28063614

[11] Stephens RJ, White SE, Cudnik M, Patterson ES. Factors associated with longer length of stay for mental health emergency department patients. J Emerg Med. 2014;47(4):412-419. doi:10.1016/j.jemermed.2014.04.040 25074781

[12] Weiss AP, Chang G, Rauch SL, . Patient- and practice-related determinants of emergency department length of stay for patients with psychiatric illness. Ann Emerg Med. 2012;60(2):162-71. doi:10.1016/j.annemergmed.2012.01.037 22555337

[13] Wharff EA, Ginnis KB, Ross AM, Blood EA. Predictors of psychiatric boarding in the pediatric emergency department: implications for emergency care. Pediatr Emerg Care. 2011;27(6):483-489. doi:10.1097/PEC.0b013e31821d8571 21629148

[14] Staggs VS. National trends and variation in nurse staffing on inpatient psychiatric units. Res Nurs Health. 2019;42(5):410-415. doi:10.1002/nur.21979 31429481

[15] O’Neil AM, Sadosty AT, Pasupathy KS, Russi C, Lohse CM, Campbell RL. Hours and miles: patient and health system implications of transfer for psychiatric bed capacity. West J Emerg Med. 2016;17(6):783-790. doi:10.5811/westjem.2016.9.30443 27833689 PMC5102608

[16] Zambrowicz R, Stewart JG, Cosby E, Esposito EC, Pridgen B, Auerbach RP. Inpatient psychiatric care outcomes for adolescents: a test of clinical and psychosocial moderators. Evid Based Pract Child Adolesc Ment Health. 2019;4(4):357-368. doi:10.1080/23794925.2019.1685419 33015362 PMC7531619

[17] Rogers SC, Mulvey CH, Divietro S, Sturm J. Escalating mental health care in pediatric emergency departments. Clin Pediatr (Phila). 2017;56(5):488-491. doi:10.1177/0009922816684609 28090789

[18] Ibeziako P, Kaufman K, Scheer KN, Sideridis G. Pediatric mental health presentations and boarding: first year of the COVID-19 pandemic. Hosp Pediatr. 2022;12(9):751-760. doi:10.1542/hpeds.2022-006555 35578918

[19] Jolicoeur L, Mullins L. Mass. physicians call on state to address ER ‘boarding’ of patients awaiting admission. WBUR. Updated February 3, 2021. Accessed May 17, 2022. https://www.wbur.org/news/2021/02/02/emergency-department-er-inpatient-beds-boarding

[20] Jolicoeur L, Mullins L. ‘This is a crisis’: mom whose son has boarded 33 days for psych bed calls for state action. WBUR News. Updated March 2, 2021. Accessed May 17, 2022. https://www.wbur.org/news/2021/02/26/mental-health-boarding-hospitals

[21] DeLaroche AM, Rodean J, Aronson PL, . Pediatric emergency department visits at US children’s hospitals during the COVID-19 pandemic. Pediatrics. 2021;147(4):e2020039628. doi:10.1542/peds.2020-039628 33361360

[22] Masler IV, Shah N, Duerring SA, Monroe KR. Effects of the COVID-19 pandemic on the pediatric emergency department: a single institution experience. Inj Epidemiol. 2022;9(suppl 1):34. doi:10.1186/s40621-022-00401-w 36544193 PMC9768778

[23] Yard E, Radhakrishnan L, Ballesteros MF, . Emergency department visits for suspected suicide attempts among persons aged 12-25 years before and during the COVID-19 pandemic—United States, January 2019–May 2021. MMWR Morb Mortal Wkly Rep. 2021;70(24):888-894. doi:10.15585/mmwr.mm7024e1 34138833 PMC8220953

[24] Duncan A, Herrera CN, Okobi M, Nandi S, Oblath R. Locked down or locked out? trends in psychiatric emergency services utilization during the COVID-19 pandemic. J Health Serv Res Policy. 2023;28(2):80-88. doi:10.1177/13558196221135119 36475326 PMC9732494

[25] Bojdani E, Rajagopalan A, Chen A, . COVID-19 pandemic: impact on psychiatric care in the United States. Psychiatry Res. 2020;289:113069. doi:10.1016/j.psychres.2020.113069 32413707 PMC7200362

[26] Janke AT, Nash KA, Goyal P, Auerbach M, Venkatesh AK. Pediatric mental health visits with prolonged length of stay in community emergency departments during COVID-19. J Am Coll Emerg Physicians Open. 2022;3(6):e12869. doi:10.1002/emp2.12869 36570374 PMC9767857

[27] Corallo B, Moreno S. Analysis of recent national trends in Medicaid and CHIP enrollment. KFF. April 4, 2023. Accessed April 28, 2023. https://www.kff.org/coronavirus-covid-19/issue-brief/analysis-of-recent-national-trends-in-medicaid-and-chip-enrollment/

[28] Sun R, Staiger B, Chan A, Baker LC, Hernandez-Boussard T. Changes in Medicaid enrollment during the COVID-19 pandemic across 6 states. Medicine (Baltimore). 2022;101(52):e32487. doi:10.1097/MD.0000000000032487 36596028 PMC9803338

[29] Guth M. State policies expanding access to behavioral health care in Medicaid. Kaiser Family Foundation. December 9, 2021. Accessed April 28, 2023. https://www.kff.org/report-section/state-policies-expanding-access-to-behavioral-health-care-in-medicaid-appendices/

[30] Kim H, McConnell KJ, Sun BC. Comparing emergency department use among Medicaid and commercial patients using all-payer all-claims data. Popul Health Manag. 2017;20(4):271-277. doi:10.1089/pop.2016.0075 28075692 PMC5564052

[31] Oblath R, Duncan A, Fortuna LR, Green JG. Racial and ethnic variations in seasonal patterns for youth psychiatric emergency department visits. J Am Acad Child Adolesc Psychiatry. 2019;58(10):S278. doi:10.1016/j.jaac.2019.08.414

[32] Oblath R, Herrera CN, Were LPO, . Long-term trends in psychiatric emergency services delivered by the Boston Emergency Services Team. *Community Ment Health J*. 2023;59:370-380. doi:10.1007/s10597-022-01015-8 PMC9399566 36001197

[33] Vakkalanka JP, Neuhaus RA, Harland KK, Clemsen L, Himadi E, Lee S. Mobile crisis outreach and emergency department utilization: a propensity score–matched analysis. West J Emerg Med. 2021;22(5):1086-1094. doi:10.5811/westjem.2021.6.52276 34546884 PMC8463043

[34] Fendrich M, Ives M, Kurz B, . Impact of mobile crisis services on emergency department use among youths with behavioral health service needs. Psychiatr Serv. 2019;70(10):881-887. doi:10.1176/appi.ps.201800450 31215355

[35] Hugo M, Smout M, Bannister J. A comparison in hospitalization rates between a community-based mobile emergency service and a hospital-based emergency service. Aust N Z J Psychiatry. 2002;36(4):504-508. doi:10.1046/j.1440-1614.2002.01042.x 12169150

[36] Currier GW, Fisher SG, Caine ED. Mobile crisis team intervention to enhance linkage of discharged suicidal emergency department patients to outpatient psychiatric services: a randomized controlled trial. Acad Emerg Med. 2010;17(1):36-43. doi:10.1111/j.1553-2712.2009.00619.x 20015106 PMC2859616

[37] Hogan MF, Goldman ML. New opportunities to improve mental health crisis systems. Psychiatr Serv. 2021;72(2):169-173. doi:10.1176/appi.ps.202000114 32988327

[38] von Elm E, Altman DG, Egger M, Pocock SJ, Gøtzsche PC, Vandenbroucke JP; STROBE Initiative. The Strengthening the Reporting of Observational Studies in Epidemiology (STROBE) statement: guidelines for reporting observational studies. Lancet. 2007;370(9596):1453-1457. doi:10.1016/S0140-6736(07)61602-X 18064739

[39] Cusick MM, Sholle ET, Davila MA, Kabariti J, Cole CL, Campion TR Jr. A method to improve availability and quality of patient race data in an electronic health record system. Appl Clin Inform. 2020;11(5):785-791. doi:10.1055/s-0040-1718756 33241548 PMC7688409

[40] Polubriaginof FCG, Ryan P, Salmasian H, . Challenges with quality of race and ethnicity data in observational databases. J Am Med Inform Assoc. 2019;26(8-9):730-736. doi:10.1093/jamia/ocz113 31365089 PMC6696496

[41] Spiranovic C, Matthews A, Scanlan J, Kirkby KC. Increasing knowledge of mental illness through secondary research of electronic health records: opportunities and challenges. *Adv Ment Health*. 2016;14(1):14-25. doi:10.1080/18387357.2015.1063635

[42] Weiskopf NG, Weng C. Methods and dimensions of electronic health record data quality assessment: enabling reuse for clinical research. J Am Med Inform Assoc. 2013;20(1):144-151. doi:10.1136/amiajnl-2011-000681 22733976 PMC3555312

[43] Alegría M, Green JG, McLaughlin KA, Loder S. Disparities in child and adolescent mental health and mental health services in the US. William T. Grant Foundation. March 2015. Accessed June 15, 2023. http://cfs.cbcs.usf.edu/projects-research/_docs/Disparities_in_child_and_adolescent_health.pdf

[44] Guessoum SB, Lachal J, Radjack R, . Adolescent psychiatric disorders during the COVID-19 pandemic and lockdown. Psychiatry Res. 2020;291:113264. doi:10.1016/j.psychres.2020.113264 32622172 PMC7323662

[45] Wong BHC, Vaezinejad M, Plener PL, et al. Lockdown stringency and paediatric self-harm presentations during COVID-19 pandemic: retrospective cohort study. *BJPsych Open*. 2022;8(2):E75. doi:10.1192/bjo.2022.41 PMC8963968 35322782

[46] Ougrin D, Wong BHC, Vaezinejad M, . Pandemic-related emergency psychiatric presentations for self-harm of children and adolescents in 10 countries (PREP-kids): a retrospective international cohort study. Eur Child Adolesc Psychiatry. 2022;31(7):1-13. doi:10.1007/s00787-021-01741-6 33677628 PMC7937052

[47] Thomas EY, Anurudran A, Robb K, Burke TF. Spotlight on child abuse and neglect response in the time of COVID-19. Lancet Public Health. 2020;5(7):e371. doi:10.1016/S2468-2667(20)30143-2 32619538 PMC7326432

[48] Samji H, Wu J, Ladak A, . Review: mental health impacts of the COVID-19 pandemic on children and youth—a systematic review. Child Adolesc Ment Health. 2022;27(2):173-189. doi:10.1111/camh.12501 34455683 PMC8653204

[49] Impact of the COVID-19 pandemic on the hospital and outpatient clinician workforce: challenges and policy responses. Office of the Assistant Secretary for Planning and Evaluation, US Dept of Health and Human Services. May 3, 2022. Issue brief HP-2022-13. Accessed February 2, 2023. https://aspe.hhs.gov/reports/covid-19-health-care-workforce

[50] Hernández-Calle D, Andreo-Jover J, Curto-Ramos J, . Pediatric mental health emergency visits during the COVID-19 pandemic. Scand J Child Adolesc Psychiatr Psychol. 2022;10(1):53-57. doi:10.2478/sjcapp-2022-0005 35836474 PMC9238432

[51] Leffler JM, Esposito CL, Frazier EA, . Crisis preparedness in acute and intensive treatment settings: lessons learned from a year of COVID-19. J Am Acad Child Adolesc Psychiatry. 2021;60(10):1171-1175. doi:10.1016/j.jaac.2021.06.016 34224838 PMC8249041

[52] Vlavianos T, McCarthy M. Positive outcomes in a virtual partial hospitalization program. Jt Comm J Qual Patient Saf. 2022;48(9):450-457. doi:10.1016/j.jcjq.2022.04.007 35660313 PMC9395212

[53] Liberati E, Richards N, Parker J, . Qualitative study of candidacy and access to secondary mental health services during the COVID-19 pandemic. Soc Sci Med. 2022;296:114711. doi:10.1016/j.socscimed.2022.114711 35063916 PMC8744250

[54] National Center for HIV, Viral Hepatitis, STD, and TB Prevention. Youth Risk Behavior Survey data summary and trends report: 2011–2021. Centers for Disease Control and Prevention. Accessed March 2, 2023. https://www.cdc.gov/healthyyouth/data/yrbs/pdf/yrbs_data-summary-trends_report2023_508.pdf

