# Supplementary material for: Psychiatric Boarding Patterns Among Publicly Insured Youths Evaluated by Mobile Crisis Teams Before and During the COVID-19 Pandemic
Source: JAMA Netw Open. 2023 Jul 6;6(7):e2321798. doi: 10.1001/jamanetworkopen.2023.21798 (PMC10326644; doi:10.1001/jamanetworkopen.2023.21798)
Supplement: Supplement 1. — eTable 1. Missing Demographic Data Compared With Study Sample eTable 2. Missing Boarding Status Compared With Known Boarding Status eTable 3. Missing Bed Search Data Compared With Known Bed Search Data [file jamanetwopen-e2321798-s001.pdf]

Supplementary Online Content

Herrera CN, Oblath R, Duncan A. Psychiatric boarding patterns among publicly insured youths evaluated by mobile crisis teams before and during the COVID-19 pandemic. *JAMA Netw Open*. 2023;6(7):e2321798. doi:10.1001/jamanetworkopen.2023.21798

eTable 1. Missing Demographic Data Compared With Study Sample

eTable 2. Missing Boarding Status Compared With Known Boarding Status

eTable 3. Missing Bed Search Data Compared With Known Bed Search Data

This supplemental material has been provided by the authors to give readers additional information about their work.

© 2023 Herrera CN et al. *JAMA Network Open*.

eTable 1. Missing Demographic Data Compared With Study Sample

		Study Sample N=7,625	Missing Demographic Data N=531	Unadjusted Comparison p-value
Time period	Pre-Pandemic	65.17%	58.19%	0.001
	Pandemic	34.83%	41.81%	
Ag	Age (Mean/SD)	13.60 (3.71)	13.73 (4.02)	0.440
Race/Ethnicity	White	19.48%	23.53%	0.720
	Black	35.74%	47.06%	
	Asian	3.79%	0.00%	
	Other	5.48%	5.88%	
	Hispanic	35.51%	23.53%	
Gender Identity	Male	47.95%	50.28%	0.020
	Female	47.29%	47.65%	
	Trans/NB	4.76%	2.07%	
English Speaking	Does not Speak English	8.97%	7.34%	0.200
	Speaks English	91.03%	92.66%	
Primary Disorder	Stress./Adjustment	47.15%	46.89%	0.630
	Psychotic	4.79%	4.71%	
	Personality	0.54%	0.19%	
	Mood	24.66%	27.68%	
	Anxiety	4.01%	4.14%	
	Developmental Delay	17.39%	15.44%	
	Eating	0.76%	0.38%	
	Other	0.71%	0.56%	
Discharge Modality	Inpatient	29.52%	33.46%	0.020
	CBAT	6.44%	6.23%	
	Outpatient	38.02%	40.27%	
	Natural Supports	26.02%	20.04%	
Boarding status	Non-Boarding	52.47%	52.35%	0.960
	Boarding	47.53%	47.65%	

Notes: Tests of significance between boarding and non-boarding samples were performed using Pearson's Chi-squared test, except for the age variable for which an ANOVA was used.

© 2023 Herrera CN et al. *JAMA Network Open*.

eTable 2. Missing Boarding Status Compared With Known Boarding Status

		Non-Boarding N=4,001	Boarding N=3,624	Missing Boarding Status N=2,645	Unadjusted Comparison to Non-Boarding Sample p-value	Unadjusted Comparison to Boarding sample p-value
Age	Age (Mean/SD)	12.99 (3.72)	14.28 (3.58)	13.03 (3.79)	0.650	0.001
Race/Ethnicity	White	14.80%	24.64%	13.12%	0.190	0.001
	Black	37.02%	34.33%	39.32%		
	Asian	3.37%	4.25%	3.25%		
	Other	5.72%	5.22%	5.22%		
	Hispanic	39.09%	31.57%	39.09%		
Gender Identity	Male	47.11%	48.87%	43.36%	0.010	0.001
	Female	49.56%	44.78%	52.85%		
	Trans/NB	3.32%	6.35%	3.78%		
English Speaking	Does not Speak English	9.50%	8.39%	9.26%	0.750	0.230
	Speaks English	90.50%	91.61%	90.74%		
Primary Disorder	Stress./Adjustment	56.04%	37.33%	59.32%	0.074	0.001
	Psychotic	2.37%	7.45%	2.16%		
	Personality	0.45%	0.63%	0.23%		
	Mood	18.75%	31.18%	17.84%		
	Anxiety	4.77%	3.17%	3.93%		
	Developmental Delay	16.92%	17.91%	15.77%		
	Eating	0.30%	1.27%	0.15%		
	Other	0.40%	1.05%	0.60%		
Discharge Modality	Inpatient	5.62%	55.91%	3.71%	0.001	<.001
	CBAT	1.75%	11.62%	1.10%		
	Outpatient	49.51%	25.33%	51.87%		
	Natural Supports	43.11%	7.15%	43.33%		

Notes: Tests of significance between boarding and non-boarding samples were performed using Pearson's Chi-squared test, except for the age variable for which an ANOVA was used.

© 2023 Herrera CN et al. *JAMA Network Open*.

eTable 3. Missing Bed Search Data Compared With Known Bed Search Data

		Missing Bed Search data N=1,872	Has Bed Search Data N=1,752	Unadjusted Comparison p-value
Age	Age (Mean/SD)	13.52 (3.30)	15.09 (3.69)	0.001
Race/Ethnicity	White	27.08%	22.03%	0.001
	Black	34.29%	34.36%	
	Asian	4.22%	4.28%	
	Other	3.63%	6.91%	
	Hispanic	30.77%	32.42%	
Gender Identity	Male	48.02%	49.77%	0.015
	Female	44.50%	45.09%	
	Trans/NB	7.48%	5.14%	
English Speaking	Does not Speak English	7.00%	9.87%	0.002
	Speaks English	93.00%	90.13%	
Primary Disorder	Stress./Adjustment	38.41%	36.19%	0.001
	Psychotic	5.82%	9.19%	
	Personality	0.80%	0.46%	
	Mood	29.86%	32.59%	
	Anxiety	3.58%	2.74%	
	Developmental Delay	19.18%	16.55%	
	Eating	1.82%	0.68%	
	Other	0.53%	1.60%	
Discharge Modality	Inpatient	42.41%	70.32%	0.001
	CBAT	11.43%	11.82%	
	Outpatient	38.41%	11.36%	
	Natural Supports	7.75%	6.51%	

Notes: Tests of significance between boarding and non-boarding samples were performed using Pearson's Chi-squared test, except for the age variable for which an ANOVA was used.

© 2023 Herrera CN et al. *JAMA Network Open*.
