# Supplementary material for: Psychiatric Boarding Patterns Among Publicly Insured Youths Evaluated by Mobile Crisis Teams Before and During the COVID-19 Pandemic
Source: JAMA Netw Open. 2023 Jul 6;6(7):e2321798. doi: 10.1001/jamanetworkopen.2023.21798 (PMC10326644; doi:10.1001/jamanetworkopen.2023.21798)
Supplement: Supplement 2. — Data Sharing Statement [file jamanetwopen-e2321798-s002.pdf]

Data Sharing Statement

Herrera. Psychiatric Boarding Patterns Among Publicly Insured Youths Evaluated by Mobile Crisis Teams Before and During the COVID-19 Pandemic. *JAMA Netw Open*. Published July 05, 2023. doi:10.1001/jamanetworkopen.2023.21798

Data
Data available: No

Additional Information
Explanation for why data not available: "The data used in this study is EMR data held by Boston Medical Center. To access the data, users are required to submit data applications and agreements in accordance with BMC policies. A de-identified data dictionary is available from the BEST Partnership for Behavioral Health, Racial, and Social Justice."
